# An Extreme Mountain Ultra-Marathon Decreases the Cost of Uphill Walking and Running

**DOI:** 10.3389/fphys.2016.00530

**Published:** 2016-11-08

**Authors:** Gianluca Vernillo, Aldo Savoldelli, Spyros Skafidas, Andrea Zignoli, Antonio La Torre, Barbara Pellegrini, Guido Giardini, Pietro Trabucchi, Grégoire P. Millet, Federico Schena

**Affiliations:** ^1^Research Center for Sport, Mountain and Health (CeRiSM), University of VeronaRovereto, Italy; ^2^Human Performance Laboratory, Faculty of Kinesiology, University of CalgaryCalgary, Canada; ^3^Department of Neurosciences, Biomedicine and Movement Sciences, University of VeronaVerona, Italy; ^4^Department of Medical and Surgical Sciences, Università degli Studi di BolognaBologna, Italy; ^5^Department of Biomedical Sciences for Health, Università degli Studi di MilanoMilan, Italy; ^6^Neurological Division, Headache Regional Centre of Aosta Valley, Regional Hospital of Aosta ValleyAosta, Italy; ^7^Institute of Sport Sciences, University of LausanneLausanne, Switzerland

**Keywords:** energy cost, running, trail, ultra-marathon, uphill, walking

## Abstract

**Purpose:** To examine the effects of the world's most challenging mountain ultramarathon (MUM, 330 km, cumulative elevation gain of +24,000 m) on the energy cost and kinematics of different uphill gaits.

**Methods:** Before (PRE) and immediately after (POST) the competition, 19 male athletes performed three submaximal 5-min treadmill exercise trials in a randomized order: walking at 5 km·h^−1^, +20%; running at 6 km·h^−1^, +15%; and running at 8 km·h^−1^, +10%. During the three trials, energy cost was assessed using an indirect calorimetry system and spatiotemporal gait parameters were acquired with a floor-level high-density photoelectric cells system.

**Results:** The average time of the study participants to complete the MUM was 129 h 43 min 48 s (range: 107 h 29 min 24 s to 144 h 21 min 0 s). Energy costs in walking (−11.5 ± 5.5%, *P* < 0.001), as well as in the first (−7.2 ± 3.1%, *P* = 0.01) and second (−7.0 ± 3.9%, *P* = 0.02) running condition decreased between PRE and POST, with a reduction both in the heart rate (−11.3, −10.0, and −9.3%, respectively) and oxygen uptake only for the walking condition (−6.5%). No consistent and significant changes in the kinematics variables were detected (*P*-values from 0.10 to 0.96).

**Conclusion:** Though fatigued after completing the MUM, the subjects were still able to maintain their uphill locomotion patterns noted at PRE. The decrease (improvement) in the energy costs was likely due to the prolonged and repetitive walking/running, reflecting a generic improvement in the mechanical efficiency of locomotion after ~130 h of uphill locomotion rather than constraints imposed by the activity on the musculoskeletal structure and function.

## Introduction

Mountain ultra-marathon (MUM) typically involves walking and running a long distance over a course with extreme changes in elevation (Millet et al., 2011; Saugy et al., 2013; Vernillo et al., 2014b, 2015a,c). During the past few years, the popularity of MUM races has grown alongside that of ultra-marathon races. Indeed, Hoffman et al. (2010) found that there was a ~5000% increase in the number of MUM races between 1978 and 2008, paralleled by an exponential growth of participation in these events, likely due to a growing appeal compared to road and track events.

It has been previously shown that after completing a 166-km (Morin et al., 2011) and a 330-km (Degache et al., 2016) MUM the subjects modified their running step mechanics and spring-mass behavior, leading to a “smoother” and “safer” running style associated with an overall lower impact. These observations have also been reported after 5 h of hilly running (Degache et al., 2013) and an uphill marathon (Lazzer et al., 2015). However, the pre-to-post evaluations in these studies were conducted under level running protocols in laboratory (Degache et al., 2013) or outdoor settings (Morin et al., 2011; Lazzer et al., 2015; Degache et al., 2016) in non-mountainous contexts that are non-ecological conditions for studying MUM (Vernillo et al., 2015c). With the aim to assess the consequences of participating in a MUM on the energy cost and kinematics of uphill locomotion, our laboratory recently showed that athletes in a fatigue state were still able to modify their uphill running pattern both after a 65-km (Vernillo et al., 2015c) and a 330-km (Vernillo et al., 2014b) MUM, with no change and a reduction (improvement) on the energy cost of uphill-running observed, respectively. These last findings challenge the notion that the metabolic cost of submaximal running at constant speeds typically drifts upwards during or after extended running exercises (Millet et al., 2009; Lazzer et al., 2012; Gimenez et al., 2013).

However, our studies investigated only the response to one uphill condition (Vernillo et al., 2014b, 2015c) and few studies have systematically examined the effect of a MUM in ecologically valid settings. Thus, it is intriguing to explore the interaction between energy cost and kinematics in determining different uphill locomotion after a MUM, where the energy demand is likely to be at the extremes of human tolerance (Millet and Millet, 2012) and the fatiguing potential high (Millet et al., 2011; Saugy et al., 2013).

To address this question, we explored the energetics and kinematics of three different uphill gaits with the purpose to determine whether fatigue-induced changes after an extreme MUM influence the energetics and kinematics of different uphill gaits.

## Materials and methods

### Participants

All runners in the MUM were invited to participate in the study via a letter prepared and sent by email by the race organizer informing them about the study purposes and design. Twenty-five male, healthy experienced ultra-trailers voluntarily participated in the pre-MUM testing protocol. Of these 25 participants, four did not complete the MUM, and one did not take part in the post-MUM testing protocol due to extreme fatigue. Another subject was physically unable to perform post-MUM testing because of hydrocele for which he was subsequently hospitalized. In all, 19 subjects underwent either the testing sessions (mean ± SD: age 43 ± 9 yrs, body mass 71.1 ± 7.5 kg, height 177.4 ± 8.0 cm, body mass index 22.6 ± 1.7 kg·m^−2^). A questionnaire was administered to collect data on EXP training experience (Vernillo et al., 2016b). On average, subjects had 12.5 ± 3.5 yrs of training in running and 5.5 ± 1.5 yrs of ultra-endurance running experience. For the year preceding the race, weekly training consisted of 3–5 sessions comprising 9.5 ± 3.5 h and 60 ± 21 km with a cumulative elevation change between 5000 and 10,000 m. All had been fully informed about the procedures and risks involved and that they could withdraw from the study at will at any time. Written informed consent was obtained from all subjects. The study was approved by the local Institutional Ethics Committee and was performed according to the ethical standards laid out in the 2013 revision of the Helsinki Declaration for experimentation on human subjects.

### Race characteristics

The international race supporting the study was the Tor des Geants® 2014. Considered the world's most challenging single-stage MUM, it entails running or walking a course of 330 km with a considerable cumulative elevation gain of +24,000 m (the sum of each elevation along the entire MUM) within 150 h. The altitude along the course ranges between 3300 and 322 m, with 20 mountain passes over 2000 m. The race is divided into seven parts interspersed by six aid-stations where athletes can rest and sleep. However, the organizing committee imposes no rules regarding rest stops, and the winner is the runner who completes the race in the shortest time, deciding on when and how long to stop for rest and feeding (for further details, see http://www.tordesgeants.it).

### Experimental design

The participants underwent two test sessions: the first was performed 1–2 days preceding the MUM (PRE) and the second immediately after the MUM (POST) in the same laboratory and location (Courmayeur, Italy, altitude 1224 m). Participants were asked to refrain from intense physical activity in the 24 h preceding PRE. Further, given the different time of the day the subjects were tested, all participants were allowed to maintain their usual diet throughout the study, but were asked to consume their last meal at least 3 h before PRE and to refrain from consuming drinks containing caffeine or alcohol for at least 12 h preceding PRE. Shortly after the subjects had crossed the finishing line, they were brought by car to the laboratory (~150 m away). Time duration between the end of the MUM and starting the POST testing procedures was <5 min. Prior to PRE and POST, body mass was measured to the nearest 0.1 kg using a digital scale positioned in the same location of the laboratory. All subjects were familiar with motorized treadmill (RunRace, Technogym, Gambettola, Italy) walking and running and wore the same trail-running shoes during the testing sessions. Before each test at PRE, subjects were instructed to perform a self-paced and self-administrated warm-up consisting of 5–10 min jogging on the treadmill. Testing was performed in random order in three different uphill gait conditions separated by 3 min rest periods: (1) a walking bout of 5 min at 5 km·h^−1^ at an inclination of +20%; (2) a running bout of 5 min at 6 km·h^−1^ at an inclination of +15%; (3) another running bout of 5 min at 8 km·h^−1^ at an inclination of +10%. The order of the conditions was randomized at PRE and identical for PRE and POST. This design was deemed appropriate on the basis of a pilot work previously conducted at our laboratory on 10 subjects that showed that the energy costs were not significantly different across conditions (iso-energetic). Condition 2 was designated as the reference condition for the iso-energetic calculation since it was the one previously investigated during this MUM (Vernillo et al., 2014b) and found to involve a “grounded running” technique (Rubenson et al., 2004). Heart rate (HR) was continuously measured and recorded during each condition with a dedicated device (RS 800 Polar Electro, Kempele, Finland). Average HR was calculated during each steady-state period. Kinematic variables were acquired using a floor-level high-density photoelectric cells system that allowed quantification of spatiotemporal gait parameters.

### Energy cost

Data were filtered into 5-s blocks for analysis. Oxygen uptake rate ($\dot{V}$ O_2_) data were sampled using the indirect calorimetry measurement (Quark CPET, Cosmed, Rome, Italy) over a 5 min period. Gas analyzer was always calibrated according to the manufacturer guidelines. Given the long-duration of the protocol, calibration of the Quark CPET metabolic system was routinely performed (including replacing the sample line and turbine between tests) so as to increase stability and sensitivity of the instrumentation (Winter, 2012). The first 4 min of each 5-min measurement were not used in the analysis in order to obtain 1 min at a flat, steady-state $\dot{V}$O_2_ plateau (Whipp and Wasserman, 1972). $\dot{V}$O_2_, carbon dioxide output ($\dot{V}$CO_2_), and respiratory exchange ratio (RER, where RER = $\dot{V}$CO_2_/$\dot{V}$O_2_) were measured using the gross $\dot{V}$O_2_ and $\dot{V}$CO_2_ measured during steady state period of each condition. The gross and net energetic of uphill walking (C_uw_), uphill grounded-running (C_ugr_), and uphill running (C_ur_) was calculated with the latter considered as the ratio between the difference in $\dot{V}$O_2_ at steady state minus $\dot{V}$O_2_ (measured at PRE for 5 min in a standing upright position) and the speed maintained during the conditions. Then, the energy expenditure in J·kg^−1^·m^−1^ was calculated by converting the $\dot{V}$O_2_ to the corresponding metabolic energy output using an energy equivalent of O_2_ ranging from 21.13 to 19.62 kJ·L^−1^ depending on the RER (Péronnet and Massicotte, 1991). The vertical cost for the three conditions (C_vuw_, C_vugr_, and C_vur_, respectively) was also assessed (Minetti, 1995; Minetti et al., 2002).

### Kinematics data acquisition

The spatiotemporal gait parameters were measured with a floor-level high-density photoelectric cells system (Optogait, Microgate, Bolzano, Italy) described and validated elsewhere (Lee et al., 2014). Briefly, this system allows the quantification of spatiotemporal gait parameters by means of a corridor of light-emitting and light-receiving diodes that are placed parallel to each other, and oriented perpendicular to the line of progression. This configuration enables the system to detect any interruption in light signal due to the presence of feet within the recording area, which is followed by the calculation of temporal features and 1D spatial coordinates of consecutive steps. The system used in this study consists of two transmitting and two receiving bars that were placed parallel to each other and 1 m apart along the longitudinal axis of the treadmill to the level of the belt, so as to collect gait parameters concurrently. Data were acquired at a sampling frequency of 1000 Hz and processed into 1D footfall patterns using the dedicated software (Optogait Software, version 1.9, Microgate, Bolzano, Italy). The test–retest reliability of this system for spatiotemporal gait parameters has been reported elsewhere (Lee et al., 2014; Gomez Bernal et al., 2016). The trial size for each condition was the minimal value, as determined from the pilot study, which would allow a reliability of 0.90 in our kinematic variables (Mullineaux et al., 2001). Accordingly, we set the value at 40 steps (20 cycles) for each condition, corresponding to no more than 1 min of registration. Data recording started 3 min after the start of each condition.

### Kinematics data processing

Data for the left and the right sides were pooled since an *a priori* analysis showed symmetrical behavior of the lower limbs during the different gaits. Kinematic variables calculated from the photocell system included: contact time (t_c_), cycle time (CT), stride frequency (SF), swing time (t_sw_ = CT − t_c_), stride length (SL), duty factor [DF = (t_c_·CT^−1^) 100]. The mean value of each variable from the recorded trials was used as a representative response and adopted in the subsequent statistical analysis.

### Statistical analysis

Data are presented as mean ± standard deviation (*SD*) and expressed in both absolute values and percentage change between PRE and POST. Results were tested for normal distribution using a Shapiro-Wilks *W*-test. Energy costs derived from the three conditions at PRE were compared using one-way ANOVA with *post-hoc* Bonferroni's procedures to determine the iso-energetic condition. Then, ANCOVA for repeated measures was performed to determine possible differences between PRE and POST MUM, with the PRE values used as a covariate. When a significant *F*-value was found, Bonferroni's *post-hoc* test was applied. The magnitude of the changes was assessed using effect size (ES) statistic with 90% confidence interval (CI) and percentage change. The ES was classified as follows: <0.2 = *trivial*, 0.2–0.6 = *small*, 0.6–1.2 = *moderate*, 1.2–2.0 *large*, >2.0 = *very large* (Hopkins et al., 2009). Pearson's product moment correlation coefficient (*r*) with 90% CI was used to examine the relationships between the percentage changes between PRE and POST in the variables and the performance time. All statistical analyses were performed using IBM™ SPSS™ Statistics (version 20.0.0, IBM Corp., Somers, NY), and the level of significance was set at α < 0.05.

## Results

The current record for the race is 70 h 4 min 15 s and the average time of the study participants was 129 h 43 min 48 s (range: 107 h 29 min 24 s to 144 h 21 min 0 s; ranking: 61st to 354th among the runners completing the race). There were no statistical differences in body mass (71.1 ± 7.5 kg vs. 70.8 ± 8.0 kg, *P* = 0.57) or body-mass index (22.6 ± 1.6 kg vs. 22.5 ± 1.7 kg, *P* = 0.54) between the PRE and POST-race sessions as well as in the energy costs derived from the three conditions at PRE (*P* > 0.05). PRE energy costs were not significantly different among the different conditions (*P* > 0.05). In general, $\dot{V}$O_2_ increased rapidly during all trials in all subjects without a discernible delay at the onset of the exercise trials and approached a plateau during the three conditions. A steady-state $\dot{V}$O_2_ was attained within 4 min for each condition without any apparent additional increase (slow component). Further, no significant correlation was found between the percentage changes between PRE and POST in the variables and the performance time (*P* > 0.05).

### Energy cost

**Figure 1:**
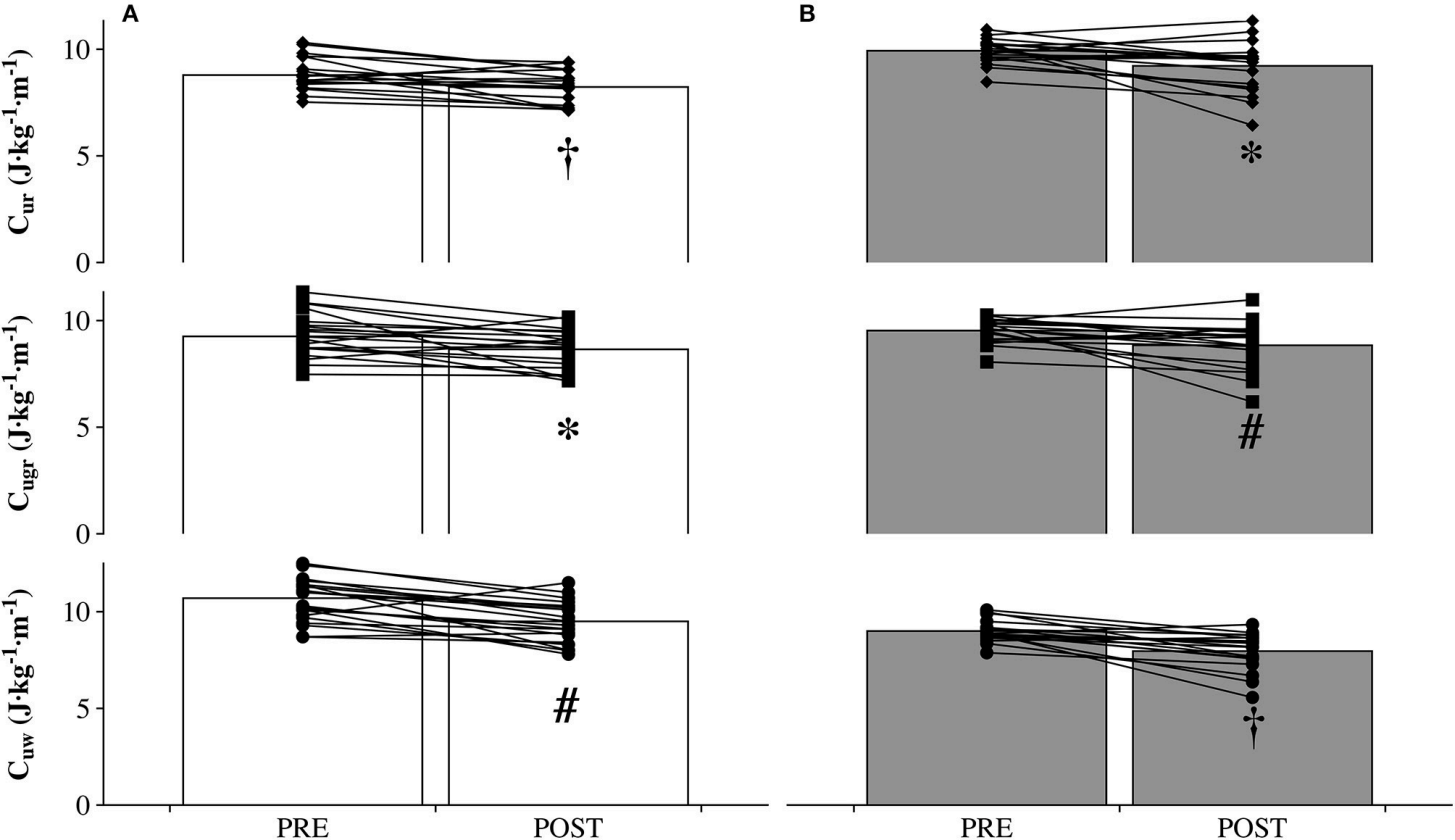
**Differences in gross (A)**, and net **(B)** energy cost before (PRE) and after (POST) the MUM (*n* = 19). ^*^P < 0.05, ^#^*P* < 0.01; †*P* < 0.001. C_uw_: Walking at 5 km·h^−1^ and +20%. C_ugr_: Grounded running at 6 km·h^−1^ and +15%. C_ur_: Running at 8 km·h^−1^ and +10%.

As shown in Table 1, $\dot{V}$ O_2_ decreased between PRE and POST for the walking condition (−6.5%, *P* = 0.004, ES = −0.83 ± 0.43, *moderate*); whereas there was a tendency toward a decrease both for the grounded running (−4.1%, *P* = 0.09, ES = −0.45 ± 0.44, *small*) and running (−3.6%, *P* = 0.09, ES = −0.40 ± 0.46, *small*) condition. $\dot{V}$CO_2_ decreased by 19.6% (*P* < 0.001, ES = −1.99 ± 0.32, *large*), 13.7% (*P* < 0.001, ES = −1.38 ± 0.41, *large*), and 14.6% (*P* < 0.001, ES = −1.31 ± 0.47, *large*) for the walking, grounded running, and running condition, respectively. RER decreased between PRE and POST for the walking (−13.4%, *P* < 0.001, ES = −1.86 ± 0.30, *large*), grounded running (−9.2%, *P* < 0.001, ES = −1.76 ± 0.55, *large*), and running (−11.0%, *P* < 0.001, ES = −2.07 ± 0.56, *very large*) conditions. HR decreased by 11.3% (*P* < 0.001, ES = −1.39 ± 0.32, *large*), 10.0% (*P* < 0.001, ES = −1.39 ± 0.32, *large*), and 9.3% (*P* < 0.001, ES = −1.30 ± 0.35, *large*) for the walking, grounded running, and running condition, respectively. Between PRE and POST, there were changes in gross C_uw_ (−10.7%, *P* = 0.001, ES = −1.06 ± 0.37, *moderate*), C_ugr_ (−6.6%, *P* = 0.02, ES = −0.64 ± 0.39, *moderate*), and C_ur_ (−7.9%, *P* = 0.01, ES = −0.73 ± 0.38, *moderate*). Net energy costs showed similar results, C_uw_ (−11.5 ± 5.5%, *P* < 0.001, ES = −1.27 ± 0.46, *large*), C_ugr_ (−7.2 ± 3.1%, *P* = 0.01, ES = −0.75 ± 0.46, *moderate*), and C_ur_ (−7.0 ± 3.9%, *P* = 0.02, ES = −0.71 ± 0.49, *moderate*; Figure 1). The vertical cost decreased by 10.4% (*P* < 0.001, ES = −1.01 ± 0.31, *moderate*), 6.8% (*P* = 0.009, ES = −0.65 ± 0.32, *moderate*), and 6.7% (*P* = 0.01, ES = −0.62 ± 0.36, *moderate*) for the walking, grounded running, and running condition, respectively (Table 1).

**Table 1:** **Changes in the metabolic data measured before (PRE) and after (POST) the MUM (*n* = 19)**.

**Variable**	**PRE**		**POST**		**% Change**
	**Mean**	***SD***	**Mean**	***SD***	
**WALKING AT 5 km·h^−1^, +20%**					
$\dot{V}$O_2_ (mL·kg^−1^·min^−1^)	41.7	2.5	39.0^#^	3.8	−6.5
$\dot{V}$O_2_ (L·min^−1^)	2.9	0.3	2.7^†^	0.3	−7.0
$\dot{V}$CO_2_ (L·min^−1^)	2.8	0.3	2.2^†^	0.2	−19.6
RER	0.93	0.06	0.81^†^	0.07	−13.4
C_vert_ (J·kg^−1^·${\text{m}}_{\text{vert}}^{-1}$)	54.0	5.0	48.3^†^	5.7	−10.4
HR (beats·min^−1^)	148.0	12.4	130.9^†^	11.0	−11.3
**RUNNING AT 6 km·h^−1^, +15%**					
$\dot{V}$O_2_ (mL·kg^−1^·min^−1^)	43.8	2.7	42.0	4.8	−4.1
$\dot{V}$O_2_ (L·min^−1^)	3.1	0.3	2.9^*^	0.3	−4.6
$\dot{V}$CO_2_ (L·min^−1^)	2.9	0.3	2.5^†^	0.2	−13.7
RER	0.94	0.04	0.85^†^	0.05	−9.2
C_vert_ (J·kg^−1^·${\text{m}}_{\text{vert}}^{-1}$)	62.5	6.6	58.0^#^	6.5	−6.8
HR (beats·min^−1^)	151.4	10.5	136.1^†^	10.3	−10.0
**RUNNING AT 8 km·h^−1^, +10%**					
$\dot{V}$O_2_ (mL·kg^−1^·min^−1^)	45.3	2.5	43.6	5.0	−3.6
$\dot{V}$O_2_ (L·min^−1^)	3.2	0.4	3.0^*^	0.4	−4.2
$\dot{V}$CO_2_ (L·min^−1^)	3.1	0.4	2.6^†^	0.3	−14.6
RER	0.95	0.05	0.84^†^	0.05	−11.0
C_vert_ (J·kg^−1^·${\text{m}}_{\text{vert}}^{-1}$)	72.7	8.0	67.5^#^	7.9	−6.7
HR (beats·min^−1^)	154.7	9.8	140.2^†^	11.4	−9.3

$\dot{V}$O_2_ (oxygen uptake rate), $\dot{V}$CO_2_ (carbon dioxide production rate), RER (respiratory exchange ratio), C_vert_ (vertical energy cost), HR (heart rate).

* P < 0.05;

# P < 0.01;

† P < 0.001.

### Kinematics data

No consistent and significant differences were detected between PRE and POST kinematics during the walking, grounded running, and running conditions (*P*-values from 0.10 to 0.96; Table 2).

**Table 2:** **Changes in the kinematics data measured before (PRE) and after (POST) the MUM (*n* = 19)**.

**Variable**	**PRE**		**POST**		**% change**
	**Mean**	***SD***	**Mean**	***SD***	
**WALKING AT 5 km·h^−1^, +20%**					
t_c_ (s)	0.679	0.042	0.666	0.042	−1.9
DF (%)	65.6	1.1	65.1	0.7	−0.7
t_s_ (s)	0.356	0.024	0.357	0.024	+0.3
CT (s)	1.036	0.062	1.022	0.064	−1.2
SF (Hz)	0.969	0.057	0.982	0.062	+1.5
SL (m)	1.44	0.1	1.42	0.1	−1.2
**RUNNING AT 6 km·h^−1^, +15%**					
t_c_ (s)	0.403	0.028	0.418	0.071	+3.7
DF (%)	53.3	3.0	53.7	5.8	+0.9
t_s_ (s)	0.354	0.032	0.357	0.045	+1.1
CT (s)	0.757	0.039	0.775	0.073	+2.5
SF (Hz)	1.325	0.070	1.300	0.112	−1.8
SL (m)	1.261	0.65	1.292	0.121	+2.5
**RUNNING AT 8 km·h^−1^, +10%**					
t_c_ (s)	0.344	0.031	0.344	0.037	+0.1
DF (%)	46.9	4.4	47.4	4.8	+1.2
t_s_ (s)	0.391	0.048	0.382	0.043	−1.7
CT (s)	0.734	0.049	0.726	0.044	−1.0
SF (Hz)	1.367	0.090	1.382	0.084	+1.3
SL (m)	1.632	0.110	1.614	0.098	−1.0

No statistical differences were detected (P > 0.05). t_c_ (contact time), DF (duty factor), t_s_ (swing time), CT (cycle time), SF (stride frequency), SL (stride length).

## Discussion

In the current study we explored the effects of the world's most challenging MUM (Tor des Geants® 2014) on the energetics and kinematics of uphill locomotion in a group of 19 experienced ultra-trailers. The main findings were: (1) C_uw_, C_ugr_, and C_ur_ decreased, paralleled by (2) no changes in the kinematic variables of the three uphill conditions.

### Energy cost

An intriguing finding of the present study was that C_uw_, C_ugr_, and C_ur_ significantly decreased after the MUM (Figure 1), paralleled by a decrease in the vertical cost (Table 1). A previous paper showed that C_ugr_ decreased after the same MUM (−13.8%) (Vernillo et al., 2014b). Therefore, although with a lower percentage change, the present data confirm this trend. The physiological determinants of energy cost include both RER and $\dot{V}$O_2_. RER at a given intensity is known to decrease with time and leads to lower caloric equivalent of O_2_, implying a decreased efficiency of ATP aerobic resynthesis and, thus, a shift in substrate utilization from carbohydrates to fats (Péronnet and Massicotte, 1991). The present study corroborates previous studies on ultra-marathon events where RER decreased between PRE and POST on a 24-h treadmill exercise (Gimenez et al., 2013), a simulated 60-km ultra-marathon (Schena et al., 2014), and 65-km MUMs (Millet et al., 2000; Vernillo et al., 2015c), likely due to a progressive depletion of glycogen stores during the MUM. But this finding differs from that previously observed by our group where RER did not change after the same MUM (Vernillo et al., 2014b). This discrepancy may be explained by the difference in time elapsed between the subjects' crossing the finishing line and their arrival at the laboratory for the POST testing procedures. In the present study, the subjects were brought directly to the laboratory (~150 m away from the finishing line), whereas in the previous study the laboratory was set up ~1 km away from the finishing line; while awaiting transport, the subjects may have understandably consumed high carbohydrate food/liquid and so were able to maintain a similar pattern of substrate utilization (authors' personal communication). The observed decrease in RER may have an obvious impact on the calculation of the energy cost, whose decrease is inevitably under- or over-estimated when the changes in the caloric equivalent of O2 are not properly considered and only the volume of O2 per unit of distance (mL·kg^−1^·min^−1^) is taken into consideration (Péronnet and Massicotte, 1991). In the present study, incorporating the RER will have influenced the calculated reductions in C_uw_, C_ugr_, and C_ur_.

However, $\dot{V}$O_2_ surprisingly decreased between PRE and POST for the uphill-walking condition, whereas a trend toward a decrease was observed both in the grounded-running and in the running conditions (Table 1). This result is in line with previous observations from a 65-km MUM (Millet et al., 2000) and is paralleled by a decrease observed in HR for the three conditions (Table 1). Even though a reduced HR has been observed after ultra-endurance exercise (Lucas et al., 2008; Mattsson et al., 2010, 2011), likely due to plasma volume expansion (Robach et al., 2014) as well as a desensitization of the heart's adrenergic receptors (Eysmann et al., 1996; Welsh et al., 2005; Hart et al., 2006), the reduced $\dot{V}$O_2_ may also indicate a lower relative exercise intensity the subjects exerted POST MUM, suggesting that specific metabolic adaptation responses occurred after an extreme MUM. This observation has been already observed after an extreme MUM (Vernillo et al., 2014b), a trekking expedition (Tam et al., 2016) as well as cycling 170 km·day^−1^ for 19 days (Slivka et al., 2012) and 2 h·day^−1^ for 5 consecutive days (Phillips et al., 1996). Although the underlying mechanism for this unexpected decrease and just how these adaptations persist over time remains unclear, the prolonged and repetitive walking/running (330 km in 1 week) may likely lead to a shift in the metabolic response of the working muscle toward a smaller imbalance between ATP supply and demand (Phillips et al., 1996), as well as positive adaptations of the oxygen transport-utilization systems (Tam et al., 2016). Taken together, these results suggest that specific metabolic adaptation responses occurred after an extreme MUM, although just how these adaptations persist over time remains unclear.

### Kinematics data

Though kinematic variables are known to potentially influence the energy cost of locomotion (Hunter and Smith, 2007), another interesting finding of the present study was that no change was noted during the three conditions (Table 1). In the present study the runners did not modify their uphill locomotion patterns, which contrasts previously studies investigating the effects of MUMs on running biomechanics (Morin et al., 2011; Degache et al., 2013; Vernillo et al., 2014b, 2015c; Lazzer et al., 2015). A possible explanation for this discrepancy may lie in the different protocols utilized. Indeed, Degache et al. (2013), as well as Morin et al. (2011) and Lazzer et al. (2015) used level running conditions that may not be ecologically valid for MUMs and inferred to uphill conditions since the mechanics of human locomotion differed when compared level to gradient locomotion (Gottschall and Kram, 2005, 2006; Vernillo et al., 2016a). However, even when uphill conditions were considered (Vernillo et al., 2014b, 2015c), no change was noted in the present study. In our opinion, the runners performed the present MUM at a slower pace compared to previous studies, and despite the fatigue and the long-lasting efforts, the mechanical alterations were not as high as those previously reported. This seems also confirmed by the fact that the changes in energetic cost are depending on the overall level of performance, and thus the locomotion speed during a MUM (Vernillo et al., 2015c). However, given also the negative elevation change experienced by the athletes in the context of this MUM (i.e., −24,000 m), future studies should also explore the effect on the kinematics and energy cost during downhill running conditions [as already done for a 65-km MUM (Vernillo et al., 2015c)], since downhill running may induce several neuromuscular, mechanical, and metabolic disturbances [for a comprehensive review see Giandolini et al. (2016)].

### Interaction between energy cost and kinematic variables

Among the many factors potentially affecting ultra-marathon performance (Millet et al., 2012), the interaction between energy cost and kinematic variables in these performances may play a significant role, though the debate on this issue continues (Millet, 2012; Millet et al., 2012). Despite the considerable muscular fatigue previously described during this MUM (Saugy et al., 2013), the subjects did not modify their uphill-locomotion characteristics. Two different mechanical methods are employed to minimize energy expenditure during terrestrial locomotion (Cavagna et al., 1977). Walking is often compared to an inverted pendulum (Mochon and McMahon, 1980); running to a spring (Cavagna et al., 1965). This implies that different hypotheses may be postulated to explain the interaction between energy cost and kinematic variables in the present study. During uphill walking the braking force is less and the propulsive force is greater (Lay et al., 2006), and this should lead to an energetically expensive propulsive impulse (Gottschall and Kram, 2003). However, because the timing of muscle activity bursts during uphill walking is shorter (Lay et al., 2007), we presume that the efficiency of muscular contraction was similar between PRE and POST MUM. Thus, the subjects were still able to walk at the freely chosen step rate, that required the least oxygen consumption (Cavagna and Franzetti, 1986). Further, it seems that the uphill stride kinematics also remained unaffected during the running conditions. However, it is worth noting that, as assessed during a level running condition, kinematic parameters as well as vertical and leg stiffness showed mixed results after a MUM (Morin et al., 2011; Degache et al., 2013; Lazzer et al., 2015; Giovanelli et al., 2016).

### Limitations

The mechanics of locomotion have not been fully evaluated. Progress has been made to measure and predict it (e.g., Morin et al., 2005; Nardello et al., 2011); however, a comprehensive investigation would require systems (e.g., 3D motion capture system and/or ground reaction forces platforms) whose application in field studies would be difficult. Analysing the energetics is still unfeasible during this MUM because it is impracticable to measure the energy cost with portable gas analysers due to logistical problems regarding the battery life and accessing the athletes. Further, it has been shown that indirect measures (e.g., armband) significantly underestimate the energy cost during uphill locomotion (Vernillo et al., 2014a, 2015b). Since a delay in the POST MUM evaluation could significantly under- or over-estimate the changes in our variables, the acute assessment immediately after may potentially give an interesting insight into the interaction between energy cost and kinematics and their adaptations during the MUM. Compared to the horizontal, uphill locomotion requires an increased work due to gaining potential energy (Higham and Biewener, 2008), thus, it may be unclear whether the adjustments observed in the present study were incline-dependent. But because we already observed that both the energy cost and the kinematics of level locomotion were not affected by this MUM (Vernillo et al., 2014b), we did not include this condition. Since the reference condition was the one previously investigated during this MUM (Vernillo et al., 2014b), we cannot exclude that the conditions applied were not the ones to minimize the energy expenditure during graded walking and running (Minetti et al., 2002; Gomeñuka et al., 2014, 2016). However, all efforts were made to reduce that bias, identifying with pilot work that the energy costs were not significantly different across conditions. Saugy et al. (2013) after the same MUM showed an increased general fatigue state associated with an increased pain at the foot and knee. Thus, pain and muscle soreness may have influenced our subjects' symmetrical locomotion. We did not measure any perceived variables associated with pain and muscle soreness because we believed that the experimental setting of the present study would not guarantee standard accuracy for collecting perceived data (Impellizzeri et al., 2011). Particularly, it was not possible to familiarize the subjects with the use of the scales. However, we tried to control that effect recording the kinematics data from 3 min into each condition, to give subjects time to be as comfortable as possible during the locomotion. Finally, even though an extreme MUM as the one from the present study leads to physiological, neuromuscular and mechanics alterations due to fatigue (e.g., Saugy et al., 2013; Vernillo et al., 2014b, 2015a; Degache et al., 2016), the non-inclusion of a control group may leave the present results susceptible to time or order effects. However, we believe that this study provides the opportunity to explore the adaptive responses of humans submitted to the extreme load and stress induced by a 330-km race with a cumulative elevation gain of +24,000 m but it must be seen as a first step toward comprehension of the physiological and biomechanics consequences of exercise as extreme as mountain ultra-marathons. Thus, further work is required to explain this interaction by means of research designs that assign participants to experimental and control groups.

## Conclusion

In conclusion, after the world's most challenging MUM the observed decrease in C_uw_, C_ugr_, and C_ur_ seems to be influenced by physiological adaptations and an improvement in the mechanical efficiency of locomotion, as already observed elsewhere (Tam et al., 2016). Indeed, uphill locomotion has a movement specific pattern (Minetti et al., 2002) and the prolonged and repetitive walking/running likely reflects positive adaptations in the neural control of the movement following ~130 h of uphill locomotion rather than constraints imposed by the activity on the musculoskeletal structure and function (Sawers et al., 2015), likely results in an improved metabolic cost. These data suggest incorporating long-lasting uphill locomotion training, and hence predisposition to sustaining such loading, in the training programs of MUM runners to optimize the training process governing this performance.

## Author contributions

Conceived and designed the experiments: GV, AS, SS, AZ, AL, BP, GM, and FS. Performed experiments: GV, AS, SS, AZ, GG, and PT. Analyzed data: GV, AS, SS, and AZ. Interpreted results of research: GV, AS, SS, AZ, AL, BP, GM, and FS. Drafted manuscript and prepared tables/figures: GV, AS, SS, and AZ. Edited, critically revised paper and approved final version of manuscript: GV, AS, SS, AZ, AL, BP, GG, PT, GM, and FS. All authors have agreed to be accountable for all aspects of the work related to its accuracy and integrity.

### Conflict of interest statement

The authors declare that the research was conducted in the absence of any commercial or financial relationships that could be construed as a potential conflict of interest.
